# Compulsive symptoms in dissociative (conversion) disorder

**DOI:** 10.4103/0019-5545.31587

**Published:** 2006

**Authors:** Arun Lata Agarwal

**Affiliations:** * Assistant Professor, Department of Psychiatry, Maulana Azad Medical College and G.B. Pant Hospital, New Delhi; *mailing address:* H-13, Mir Dard Lane, MAMC Campus, New Delhi 110002; e-mail: dralagrawal@hotmail.com

**Keywords:** Obsession–compulsion, conversion–dissociation, neurosis

## Abstract

According to Mayer-Gross, Slater and Roth's classical textbook *Clinical psychiatry,* obsessive-compulsive symptoms are rarely seen in hysteria. The release of obsessive-compulsive symptoms is said to occur only in those who are constitutionally predisposed. In this context, the case of a young woman with dissociative (conversion) disorder, who presented with compulsive symptoms, is reported. In her case, the dissociative phenomena manifested as compulsive symptoms without concomitant predisposing factors. Management on the line of treatment for hysteria promptly achieved lasting resolution of symptoms without recourse to pharmacological or non-pharmacological treatment strategies used for obsession(s) or compulsion(s). The underlying mechanism(s) are discussed.

## INTRODUCTION

According to conventional clinical teaching, obsessive–compulsive symptoms (OCS) are rarely seen in the dissociative (conversion) disorder (DCD). This fact is also reflected in the statement mentioned in a classical textbook: ‘compulsive symptoms are… rare… in hysterical reactions’, which further adds that OCS are released only in those who are consti-tutionally predisposed and not otherwise.^1^ In contrast, DCD can occur even without a predisposing medical, neurological or psychiatric disorder.^2^

The robustness of the above-mentioned clinical wisdom is borne out by the sparseness of published literature in this area. Kaminsky and Slavney have published two reports dealing with prominent OCS and obsessional traits in patients with the briquet syndrome^3^,^4^ and hysterical personality.^4^ Bieniecka and Sulestrowska reported the case of a child whose compulsive motor acts were hysterical reactions caused by an insurmountable fear of school.^5^ Ross and Anderson observed ego-alien OCS-like symptoms in many patients suffering with multiple personality disorder.^6^ No Indian data on compulsive symptoms in hysterical reactions have been published. One such case is reported below and the probable underlying mechanisms have been discussed.

## THE CASE

Ms K, a 19-year-old matriculate and housewife of rural origin from a low socioeconomic status and married for 10 months, was brought by her spouse and in-laws, with problems of 3 days' duration. The history revealed that within a month of marriage K began refusing sex. In the ensuing 3 months she often quarrelled after sex and even impulsively attempted suicide thrice.

During the next 4 months, K's unwillingness for sex continued, along with recurrent amnesia of marriage. Additionally, she would be possessed, up to 5 times a day, by the ‘spirit’ of a young female (of M, who, though a postgraduate, had committed suicide because of failure in love). The spirit of M claimed: ‘I had entered K 3 days prior to her [i.e. K's] marriage. Therefore marriage was with me, and K, being unmarried, should not be troubled [i.e. sexually] by the husband.’ When faith-healing did not give lasting relief, village elders reorganized their marriage rites on 3–4 occasions, but each time the improvement, including permitting sex, was limited to 2–3 weeks only. Thereafter, K again became quarrelsome, gave suicide threats and demanded to be sent to her natal place, where she remained well for a month. Her reluctance for sex persisted even after returning from her natal place and during the following one month she had four pseudoseizures after sex.

Ms K again had sex with her husband, few days after the last pseudoseizure. Immediately thereafter, she developed the symptoms of repeated hand-washing and bathing, not touching anything besides not eating, drinking or talking. These symptoms resembled her husband's obsession regarding contamination (by dirt) and compulsive hand-washing, which he had developed a few months after their engagement, but which he had completely remitted within 3 months of treatment with fluoxetine prescribed by a psychiatrist elsewhere. Subsequently, he had remained well.

The appearance of similar symptoms in K, immediately following sex, prompted speculation among filial relatives that the husband's past illness may have been (sexually) contagious, and that she may also need similar medical treatment. So K was brought directly to a psychiatrist, the first physician to be contacted after marriage, within 3 days of the onset of these symptoms.

At the time of consultation, the results of K's physical examination were within normal limits and her mental state revealed mutism, anxiety and compulsive hand-washing. In view of her 9-month history preceding the consultation, for seemingly compulsive behaviour, K was diagnosed and treated as a case of mixed dissociative (conversion) disorder (F 44.7), according to the ICD-10.

The presenting symptoms ameliorated within 24 hours of admission (due to the change of environment) as a result of an initial transfusion of a bottle of dextrose saline (used as psychological symbol of medical treatment), and encouragement for her to express herself. Simultaneously, the family members' cooperation was solicited, their misconceptions regarding the contagious nature of illness dispelled, their other anxieties allayed and psycho-education imparted.

### Reconstruction of the background

Information gathered primarily from K, and supplemented by her husband and in-laws, suggested that premorbidly K had prominent histrionic traits not amounting to a personality disorder.

K was engaged by her parents at the age of 16 years, on the personal initiative of the would-be bridegroom (i.e. her current husband), much against her wish. In spite of the groom's desire for early marriage, she had delayed it for more than 2 years.

A few months before engagement, K had felt severely disappointed on (forced) discontinuation of her studies (beyond matriculation) as per the village norm for girls of her age. During this time, her brother-in-law (i.e. her sister's husband) was quite supportive of her and gradually became physically intimate with her. Their clandestine sexual relationship continued, sporadically, for long even after her engagement till she terminated it after much procrastination, and consented for marriage. However, her emotional turmoil continued on account of unresolved grief over the loss of a relationship and guilt of premarital sexual relations with another man.

The consummation of marriage further compounded her emotional dissonance, which she could not contain despite her best efforts. She soon developed marked irritability and severe disinclination towards sex. Her husband, although quite supportive, bore the major brunt, for none of his fault, which further burdened her. Therefore, after brief period(s) of sexual abstinence, she reluctantly but repeatedly gave in to her husband's urgings for sex. In the ensuing emotional upheaval, however, the afore-mentioned problem behaviours appeared one after the other.

Immediately following sex, the last time before presentation, K was suddenly afflicted by a queer thought that she was contaminated by ‘semen’, and that she was even spreading the contamination through her hands. She was unable to control these thoughts, which started to increase in her husband's presence. Her thoughts were her so uncontrollable, senseless, repetitive and distressing that she had to repeatedly wash her hands, bathe or change her clothes; she could not touch anything, as any touched object also had to be washed repeatedly. Moreover, she could not eat or drink anything, as any touched food or drink also had to be discarded. To make matters worse, the nature of her thoughts precluded their sharing, and this led to her mutism.

Once K had emotionally unburdened herself, only the conscious conflicts were addressed in individual and conjoint therapy sessions, along with efforts to augment her coping skills. She gradually came to terms with her marriage, was able to adjust well with her husband, and continued to be asymptomatic till 9 months of follow-up, after which further visits were discontinued on request.

## DISCUSSION

Viewed socioculturally,^2^ the patient's distress signals did communicate her cry for help, in addition to achieving temporary avoidance of the distressing situation (i.e. marital sex). Her spouse and in-laws also recognized her cries for help, however, not as a ‘medical’ problem but on the lines of a shared sociocultural belief system, which is further borne out by the husband's statement: ‘We followed the advice rendered by the family and village elders, priests, *ojhas* and *fakirs,* etc. for her “other” problems for almost 9 months continuously but we brought [her] to a medical doctor [a psychiatrist] when she developed an actual illness, similar to my earlier disease. We suspect that she got it from me.'

Viewed psycho-dynamically,^2^ the ongoing conflicts unconsciously reactivated a (related) latent intrapsychic conflict, necessitating deployment of symptom-forming defences, so as to lessen the intrapsychic anxiety. However, the presence of (conscious) anxiety, in spite of these defences, in such patients is in accordance with the literature.^7^,^8^ The presence of obvious elements of personal significance (of the recent past) in the patient's symptomatology is also in accordance with the literature.^1^

The most valid definition of OCS is said to include the following three elements: intrusiveness, repetitiveness, and groups of resistance, distress, irrationality and difficulty in dismissing.^9^ The presenting complaint had all these three elements and thus qualified as an OCS.

Still, in this case, the manner in which these OCS suddenly appeared, in the absence of premorbid traits of obsessionality, following a recurring precipitant (sex with husband), for brief duration (4 days), their superficial resemblance with the husband's past illness (conversion model), their exacerbation in the husband's presence and their rapid resolution without specific pharmacological or behavioural intervention directed at them suggests that these OCS were generated not by the classical mechanisms of a true obsessional but by the same mechanisms which had brought about other DCD. Ross and Anderson have also argued that auto-hypnotic DCD patients are capable of exhibiting diverse pheno-menology of different diagnoses and, in such cases, all the diverse presentations should be viewed as facets of a single polysymtomatic DCD rather than comorbid diagnoses.^6^

### Practice point

Despite the avowed rarity of OCS in DCD, this case illustrates the importance of underlying diagnosis even when clear OCS are present. The history of illness and the context of symptoms provide important clues to diagnosis. The OCS in these patients respond to the same treatment strategies as used in the management of DCD.
